# Raising Awareness for Sustainable Faecal Treatment Using Augmented Reality

**DOI:** 10.3390/ijerph21121634

**Published:** 2024-12-08

**Authors:** Yurina Otaki, Hidehito Honda, Yutaro Onuki, Gen Shinohara, Masahiro Otaki, Tushara Chaminda

**Affiliations:** 1 Graduate School of Social Science, Hitotsubashi University, 2-1 Naka, Kunitachi-shi, Tokyo 186-8601, Japan; yu0302onuki@gmail.com (Y.O.);; 2 Faculty of Pychology, Otemongakuin University, 2-1-15 Nishiai, Ibaraki-shi, Osaka 567-8502, Japan; hitohonda.02@gmail.com; 3 Faculty of Core Research, Ochanomizu University, 2-1-1 Otsuka, Bunkyo-ku, Tokyo 112-8610, Japan; otaki.masahiro@ocha.ac.jp; 4 Faculty of Engineering, University of Ruhuna, Hapugala, Galle 80000, Sri Lanka; tusharac@cee.ruh.ac.lk

**Keywords:** augmented reality-based teaching, contamination by human excreta, environmental and health problems, human faecal treatment, low- and middle-income countries, pit latrines, septic tanks

## Abstract

Pit latrines—the simplest on-site sanitation system—have been extensively used in developing countries in Asia for a long time. However, pit latrines are pollution and health risk hotspots that can cause widespread contamination. It is preferable to upgrade them to septic tanks, which are more advanced, effective, and simple alternatives. This study encourages the transition from pit latrines to septic tanks by making people aware of the health and environmental risks associated with the use of pit latrines. As decisions about sanitation technologies are mostly made by individual households, it is important to find communication tools for the average household to understand the basic information to make informed decisions. To this end, this study used augmented reality as a communication tool. A survey was conducted with Sri Lankan households that use pit latrines, and experiments were carried out in a university laboratory in Japan. The use of augmented reality increased people’s understanding of the environmental and health risks of pit latrines. This understanding was retained for some time because people found the use of augmented reality ‘enjoyable’. Hence, our findings contribute to the promotion of the transition from pit latrines to septic tanks in low- and middle-income countries.

## 1. Introduction

Target 6 of the United Nations Sustainable Development Goals (SDGs) is to ensure the availability and sustainable management of water and sanitation for all. Safely managed sanitation services, as set out in SDG 6.2, present a challenge that focuses on comprehensive and safe excreta management. This goal extends beyond preventing human contact with excreta, adopting a holistic approach for the entire process, that is, from the first stage of treatment to the discharge of the properly treated matter into the environment [1]. In most low- and middle-income countries (LMICs), efforts to improve water supply are ongoing; however, they have not kept pace with sanitation improvements. Therefore, the sanitation sector is lagging [2]. As inadequate and ineffective sanitation measures remain a major risk to global public health—posing harmful effects, such as diarrhoeal deaths, especially among young children [3]—sanitary conditions in LMICs need to be improved as soon as possible.

Currently, on-site sanitation systems (OSSs), such as septic tanks and pit latrines, are predominantly used in LMICs for faecal treatment [4]. Although pit latrines, the simplest OSS, are considered the sanitation technology of choice in low-income settings, they are pollution and health risk hotspots [5]. Pit latrines, originally not designed to be used as flushing toilets, spread contamination when connected to flushing toilets [6]. Therefore, it is preferable to move up the sanitation ladder to septic tanks, which are a more advanced and easier-to-use alternative [2].

This study encourages the transition from pit latrines to septic tanks by making people aware of the health and environmental risks associated with pit latrine usage. As individual households make decisions regarding the OSS used (i.e., a pit latrine or a septic tank), it is necessary to communicate the need for and benefits of the transition to the affected public in an easily comprehensible manner. This study examines the means of information communication that support people in LMICs in understanding the practical details of OSSs and accurately identifies their problems to motivate them to make the switch. Tokunaga et al. [7] used a comparison of still images and videos to communicate the hygiene risks of pit latrines and encourage the transition to septic tanks. They reported that the video material increased people’s preference for switching to septic tanks, with more than 80% of the participants wanting to install septic tanks in the future. However, they failed to change their perception of health and environmental risks concomitant with the use of pit latrines. As the comprehension of the environmental and health issues associated with pit latrines is fundamental to encourage the widespread use of septic tanks, we attempted to utilise augmented reality (AR) to graphically demonstrate the real environment using virtual objects (computer graphics) [8,9].

AR can be used to help people experience and understand things that are difficult to experience and observe in real-life situations [10], such as the human digestive system [11] and plant growth [12]. As pit latrines are installed underground and entail the functioning of microorganisms that are invisible to the human eye, it may be difficult to imagine and understand the environment and health risks. Therefore, AR was employed in this study to facilitate people’s understanding of the subject. The results showed that AR-based communication helped people accurately identify the environmental and health impacts of pit latrines because the presentation of the information made viewing the material more enjoyable.

## 2. Materials and Methods

### 2.1. Effectiveness of AR in Communicating Information

This study was conducted in Sri Lanka, where approximately 80% of households use pit latrines [13]. The department with jurisdiction over wastewater treatment recommends avoiding pit latrine usage and constructing properly designed septic tanks [14].

#### 2.1.1. Survey Design

A survey was conducted with households using pit latrines. Survey participants were recruited using snowball sampling. The participants were asked how they felt about the environmental and health impacts of pit latrines that they were currently using. We explained to them how toilet wastewater is treated in pit latrines and septic tanks, the different residence times, maintenance requirements, and problems that can arise via textual information. This was followed by a detailed explanation of the sanitary problems caused by pit latrines in areas of clustered housing, using either video or video plus AR. Immediately after receiving these explanations, they were quizzed about pit latrines and septic tanks (residence time, maintenance requirements, maintenance details, and environmental and health risks) to ascertain their understanding levels. Following this, they were again asked how they felt about the environmental and health aspects of their pit latrines. Two weeks later, they were asked to answer the same quiz and questions to assess their retention and perceptions. Memory retention consists of four stages—working memory for the first 60 s, early long-term memory lasting from 60 s to 12 h, transitional long-term memory lasting from 12 h to 1 week, and long-lasting memory [15]. Therefore, we hypothesised that by asking the question again two weeks later, we would capture the first stage of long-lasting memory.

The textual information and videos used for the explanations were the same as those used by Tokunaga et al.’s study [7] and are provided as Supplementary Materials (Figures S1 and S2). This study was approved by the Hitotsubashi University Ethical Committee (D019).

#### 2.1.2. AR Interventions

**Figure 1:**
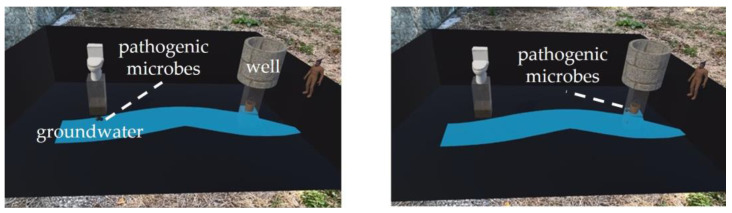
Screenshots from the AR. This shows the movement of pathogenic microbes from the toilet (**left** picture) through groundwater to the well (**right** picture).

To illustrate the potential negative impact of wastewater from pit latrines on human health and the environment, a three-dimensional (3D) animation was used in AR to demonstrate how wastewater from the toilet flowed through the pit latrine into the groundwater. The 3D models of the toilet, pit latrine, groundwater, and well were made using the 3D-CG creation software Blender 3.5 (https://www.blender.org/, accessed on 12 March 2023). The animation was added to the 3D models using the game engine Unity 2021.3.21f1 (https://unity.com/, accessed on 12 March 2023), while the completed 3D animation was viewed as AR on an iPad. The animation lasted approximately 10 s and was programmed to loop, restarting automatically upon completion. Screenshots from the AR are presented in Figure 1 and Supplementary Materials.

When presenting the AR, we read the following information to the participants: “This presentation demonstrates your pit latrine and the surrounding groundwater. When you and your family defecate and flush the toilet, the excreta go into the pit latrine. After that, it seeps from the pit latrine little by little into the soil and eventually reaches the groundwater. Therefore, when a sick person goes to the toilet, the excrement can reach and contaminate the nearby well via the pit latrine”.

**Figure 2:**
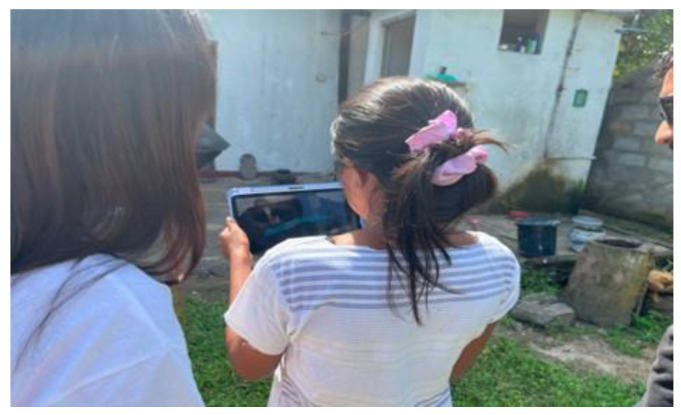
Presenting AR materials to the participants using iPads.

AR materials were presented to the participants using iPads at the location of the pit latrine to provide a sense of AR (Figure 2). The participants were able to manipulate the screen to view the 3D model from various angles, with the model adjusting in real-time according to the device’s movement.

#### 2.1.3. Assessment of the Impact of the Intervention

Participants were asked three times about their perceptions regarding the environmental and health risks of their pit latrine, including at the beginning of the survey (‘before_environment’ and ‘before_health’, respectively), immediately after they were explained about the negative impacts of pit latrines (‘after_environment’ and ‘after_health’, respectively), and two weeks later (‘later_environment’ and ‘later_health’, respectively). Their responses were recorded on a five-point Likert scale, with 1 = problematic, 2 = somewhat problematic, 3 = undecided, 4 = not very problematic, and 5 = not problematic.

We performed ordinal logistic regression analysis on the obtained data using the following model. For the dependent variable, we used the ratings of their perceptions immediately after the intervention (‘after_environment’ and ‘after_health’, respectively) and two weeks after the intervention (‘later_environment’ and ‘later_health’, respectively). For the independent variables, we used the ratings of their original perception (‘before_environment’ and ‘before_health’, respectively) and the number of correct answers in the quiz about pit latrines and septic tanks immediately after the explanation (quiz). The independent variables were combined linearly. We assumed a hierarchical structure for the parameters, while the weights for the independent variables and the four rating criteria were estimated using hierarchical Bayesian parameter estimation.

In the hierarchical Bayesian framework, we assumed that group *i* had its own parameter for each variable and intercept. The groups were structured based on two interventions (i.e., AR + video and video only) and two age categories (younger and older). These two factors were crossed to form four distinct groups. Regarding the age categories, the sample was divided into two groups using the median age (49 years) as the cutoff. Respondents with ages equal to or below the median were classified as ‘younger’, while those with ages above the median were classified as ‘older’. We assumed that each parameter came from a group-level normal distribution, N(μ_{all}, σ^2^). For μ_{all} and σ^2^, we used uninformative priors, μ_{all}~U(−50, 50) and σ^2^~U(0, 150). The parameter estimation was conducted using Stan, implemented through the R library cmdstanr.

### 2.2. Verification of Factors Contributing to the Effectiveness of AR

**Figure 3:**
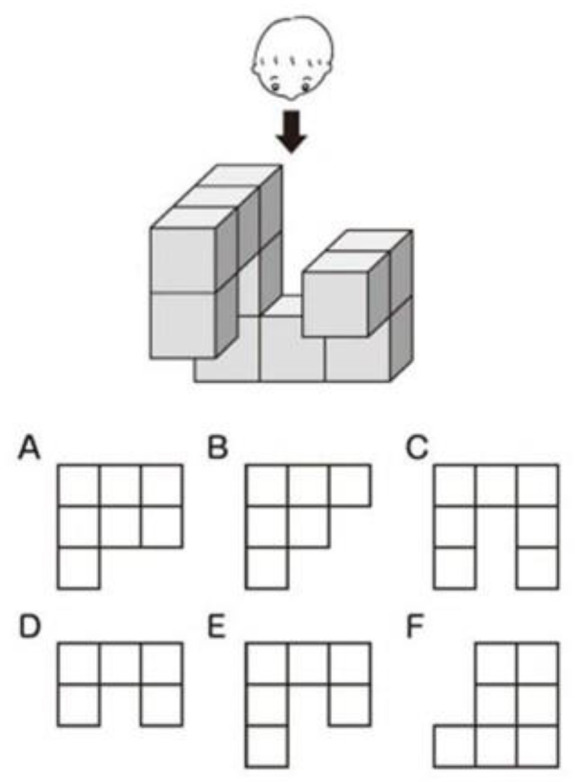
Mental rotation task.

An experiment was conducted to test AR’s effectiveness when the participants were provided with information about OSSs. Experiments were conducted in a university laboratory in Japan and presented as an illustration of toilet effluent treatment in developing countries in Asia. The participants were asked to perform a mental rotation task to assess their thinking and cognitive functions (Figure 3). The same explanation as that in Section 2.1 was presented to them. They were divided into two groups—one group was presented with the same AR as that used in the survey in Sri Lanka (Group 1), while the other group was presented with a 3D animation video that displayed the same model as used in the AR (Group 2). Each group had 30 participants, with an even number of participants in their 20s, 30s, 40s, 50s, and 60s or older.

After the intervention, the participants were asked to rate their impressions of the material using a visual analogue scale with seven items—(1) their impressions regarding toilet conditions in developing countries in Asia (0 = not impressed at all to 100 = very impressed), (2) reality regarding toilet conditions in developing countries in Asia (0 = no sense of reality at all to 100 = a very strong sense of reality), (3) receptiveness to knowledge about toilet conditions in developing countries in Asia (0 = not willing to gain knowledge at all to 100 = very willing to gain knowledge), (4) willingness to learn more about toilet conditions in developing countries in Asia (0 = not interested in learning at all to 100 = very interested in learning), (5) implications of the pit latrine system (0 = could not picture it concretely at all to 100 = could picture it very concretely), (6) concentration when viewing the material (0 = could not concentrate at all to 100 = fully concentrated), and (7) enjoyment of the material (0 = did not enjoy it at all to 100 = enjoyed it very much).

Question: Which of these three-dimensional figures looks correct when viewed from the direction of the arrow (directly above)?

## 3. Results

### 3.1. Effectiveness of AR in Communicating Information Regarding Environmental Aspects

**Figure 4:**
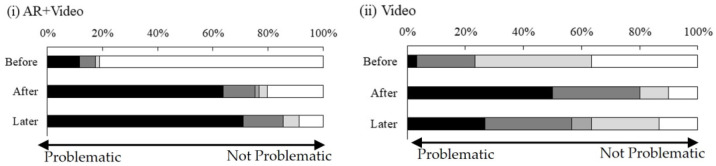
Perception of the impact of pit latrines on the environment.

Regarding the assessment of the environmental impact of pit latrines immediately after the intervention (after_environment) and two weeks after the intervention (later_environment), a smaller value indicated that the participants considered the impact to be more problematic; that is, the participants were aware of the concomitant problems of pit latrines. Compared with the video-only condition, the AR + video condition led to a more widespread perception among the participants that the impact of pit latrines on the environment was problematic and they exhibited continued awareness of the problem two weeks after the intervention (Figure 4).

Table 1 presents the results of the ordinal logistic regression analysis regarding the intervention’s impact. A low rating indicates a relatively correct understanding of the negative environmental impact of pit latrines. That is, a lower estimated parameter indicates a relatively accurate understanding of the negative environmental impact of pit latrines. As such, the participants were more likely to accurately perceive the environmental impact of pit latrines when the AR was presented immediately after (after_environment) and two weeks after the intervention (later_environment). However, its effects varied by age group.

The participants’ initial perception (before_environment) did not influence the environmental assessment immediately after the intervention in the younger group (95% CI contained 0); however, it did have an impact two weeks after the intervention (95% CI did not include 0). As for the older group, a different trend was observed; initial perception (before_environment) impacted the older group both immediately after and two weeks after the intervention (95% CI did not include 0). Furthermore, while the participants’ correct knowledge (quiz) affected the environmental assessment immediately after and two weeks after the interventions for the younger group with AR intervention (95% CI did not include 0), this did not occur in the case of the video only condition (95% CI contained 0). Regarding the older group, a different trend was observed. It was impacted immediately after the intervention (95% CI did not include 0), but not two weeks after the intervention, regardless of the type of intervention.

**Figure 5:**
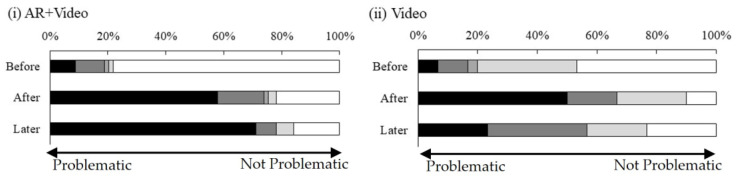
Perception of the health impact of pit latrines.

Regarding the assessment of the participants’ perceptions of the health impact of pit latrines, compared with the video only condition, the AR + video condition led to a more widespread perception that the impact of pit latrines on health was problematic. The participants exhibited continued awareness of the problem immediately after and two weeks after the intervention (Figure 5).

Table 2 presents the results of the health impact analysis. As with environmental impact, a low rating also indicates a relatively correct understanding of the negative health impact of pit latrines. That is, a lower estimated parameter indicates a relatively accurate understanding of the negative health impact of pit latrines. Thus, the results of the ordinal logistic regression analysis can be summarized as follows.

The results differed in several aspects from that of the environmental assessment. The participants were more likely to accurately perceive the health impact of pit latrines when the AR was presented immediately after the intervention (after_health) for all, and two weeks after the intervention (later_health) only for the older generation. In both age groups, immediately after the intervention, the effect of the initial perception (before_health) was observed (95% CI did not include 0); however, after two weeks, its influence became weaker (95% CI did not include 0). Regarding the acquisition of correct knowledge (quiz), it impacted the health assessment immediately after the intervention (95% CI did not include 0) for both generations, but it did not have any influence two weeks after the intervention (95% CI contained 0) except in the case of the AR intervention for the younger generation.

### 3.2. Verification of Factors Contributing to AR Effectiveness

**Figure 6:**
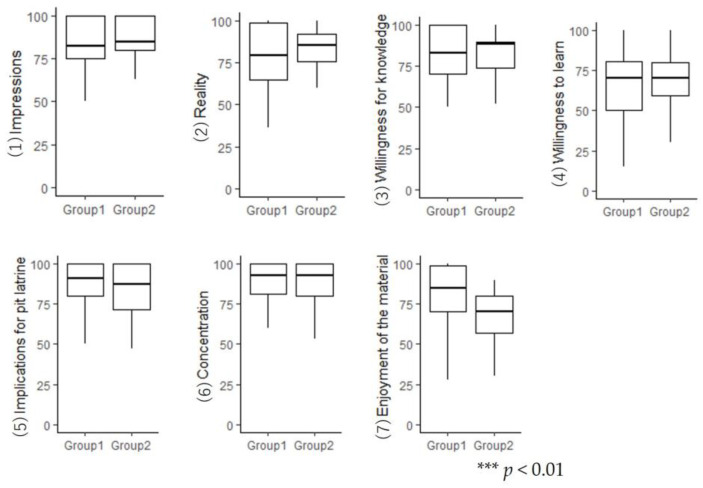
Impressions of the information presented.

There were no significant differences in the percentages of correct responses to the mental rotation task between the groups (*W* = 420, *p* = 0.458, *r* = 0.14). Therefore, the participants’ thinking and cognitive functions did not differ between the groups; hence, both groups were considered equally fit for subsequent analysis. Regarding the participants’ impressions of the information presented, only item 7 (‘enjoyment of the material’) showed a significant difference (Table 3). Participants in Group 1, who experienced the AR feature, were significantly more likely to find the material ‘enjoyable’ (Figure 6).

## 4. Discussion

AR was effective in helping the participants accurately assess the environmental and health impacts of pit latrines; however, its effectiveness varied by age group. Younger participants discarded their original perceptions, especially regarding environmental impact, immediately after the AR intervention and their perceptions changed based on the information conveyed via AR. Conversely, older participants’ original perceptions did not change immediately; they changed a short time after. Prior studies have noted that older adults respond differently to AR, compared with younger adults, because cognitive function declines with age [16]. Older participants are significantly slower when using AR, compared with younger participants; however, their error rates are not significantly different [17]. Regarding the subject of pit latrines, the effect of age was not a negative factor because it did not involve an immediate decision with high frequency (e.g., shopping for daily necessities); instead, it involved a transition to a new system after careful consideration.

As AR is effective in both physical and virtual learning [18] and can help improve spatial capabilities [19], it helps people understand invisible mechanisms. AR-based learning is more likely to be retained in people’s long-term memories than learning via video- or paper-based media [20,21,22]. In this study, the information provided using AR continued to be recognised and understood by the participants a fortnight after its presentation. In addition, as novel media enhance short- and long-term recall [23], AR was a novel experience for the Sri Lankan population, which contributed to its effectiveness. This supports studies that reported that AR had an effective communication impact due to its novelty and enjoyment [24], which worked positively to deepen the understanding in this study. As AR augments real space, the feature of experiencing it in situ may be effective. In this study, the participants experienced AR by moving to a location with a pit latrine. It has been noted that an actual action is more likely to be remembered [25] than theoretical information, and learning is improved when relevant information is presented spatially or temporally close to the subject [26].

We presented AR using an iPad so that all the participants had the same experience. However, because AR is an application, it can be displayed with a smartphone as well. Smartphone ownership is increasing in developing countries. It has been pointed out that income level does not affect smartphone ownership in developing countries unlike in developed countries [27]. Therefore, AR interventions can be an effective tool for various populations in developing countries.

Previous research has indicated that an augmented sense of reality is effective during teaching and learning [28]. However, in the present study, the sense of reality was not enhanced by AR. AR contributes to high motivation [29,30]; it can be one of the reasons for the participants to consider AR to be ‘enjoyable’.

This study had certain limitations. Given that OSSs are home facilities, a switch to alternatives does not occur overnight. It usually takes years or even decades before a decision regarding such changes is made. Therefore, it is seldom possible to confirm whether the desired change is reflected in the participants’ actual behaviour. In the future, it would be desirable to conduct a more comprehensive evaluation of the AR’s impact on the real world through long-term studies using actual behavioural changes, such as the adoption of septic tanks, as an indicator. The number of participants was limited and the participants were recruited through snowball sampling, which may have resulted in bias. Snowball sampling is particularly suited for surveys in hard-to-reach populations or within specific networks. Therefore, the sample chosen is not representative of the population, and the generalisation of results requires caution. However, as there are no research companies in LMICs, it is not always easy to secure sufficient survey targets. In the future, more generalised conclusions can be drawn by revising the sampling methodology and conducting field surveys with a larger sample. Owing to the insufficient experimental resources for this study, we were unable to establish a control group. Therefore, we were unable to make a comparison with the group with no intervention, and thus could not independently measure the effect of the intervention. Further findings, including comparisons with the control group, are necessary in the future. However, while there was no evidence that the intervention itself had any negative impact, and educational effects were observed before and after the intervention, this study’s results indicate the great potential of educational interventions using AR technology. AR quality was an issue. The AR used in this study was simple; however, if higher-quality AR is used, the factors that affect participants’ behaviour may change. For example, future studies should add manipulation elements, such as zooming in and out, to enhance reality augmentation and show the differences when using the same amount of information as a 3D animation. However, it should be noted that complex AR may increase cognitive load and weaken its effectiveness [31]. For effective AR, it is important to have a user-friendly interface that is easy to use even for users unfamiliar with technology [32]. This should be taken into consideration when developing future AR. Although age was considered as a moderator in this study, other potential factors such as education level, income, and experience with AR technology should also be considered in the future. However, these may have a smaller impact on the results than the factors considered in this study [33]. Furthermore, respondents were asked to self-report their perceptions of environmental and health risks, which may lead to answers that they consider socially desirable. Therefore, the data may be subject to response bias. However, as this study examines the AR effect by comparing two groups and considering the possibility of response bias in both groups, the differences obtained are considered valid. Additionally, we have to measure participant engagement or interactivity with the AR in the future.

## 5. Conclusions

In this study, AR was used to explore a means of information communication that supported people in LMICs in accurately identifying the environmental and health problems of using pit latrines. The results showed that AR-based information transfer helped people accurately identify the environmental and health impacts of pit latrines. This is because the presentation of information made the viewing experience enjoyable. Compared with younger individuals, older participants took longer to change their minds. However, this is not a major issue because a long period is involved when considering changing an OSS from a pit latrine to a septic tank.

The finding that AR increased the understanding of OSSs is significant as it points to the possibility of contributing to the promotion of this transition in LMICs. Although the intervention experiment was conducted with the belief that accurate problem recognition would promote the transition, further careful consideration is needed to determine whether such a perception change will lead to the actual introduction of septic tanks. This study identified the effects and challenges of each AR element, which can be useful references for future AR-based information dissemination.

In the future, longitudinal studies can be conducted to evaluate the lasting effects of AR and explore contextual factors that may influence the effects of AR through field experiments in various cultures, leading to social implementation. A proactive involvement of local authorities is essential to fully provide such information to the residents. However, local authorities in developing countries lack the knowledge and capacity regarding human waste treatment [34]. Hence, the education of local authorities is essential for effective social implementation.

## Figures and Tables

**Table 1:** Results of parameter estimations when the dependent variable comprised the rating of the participants’ perceptions of the environmental impact of pit latrines.

Variable	Age	Intervention	After_Environment		Later_Environment	
			Median	95% CI	Median	95% CI
before_environment	Younger	AR + Video	0.217	(−0.123)–(0.572)	0.614	**(0.164)–(1.145)**
		Video only	0.338	(−0.013)–(0.732)	0.678	**(0.235)–(1.256)**
	Older	AR + Video	0.420	**(0.124)–(0.795)**	0.562	**(0.174)–(1.056)**
		Video only	0.473	**(0.133)–(1.044)**	0.763	**(0.319)–(1.492)**
quiz	Younger	AR + Video	−0.618	**(−1.284)–(−0.053)**	−0.614	**(−1.313)–(−0.057)**
		Video only	−0.577	(−1.263)–(0.036)	−0.073	(−0.710)–(0.650)
	Older	AR + Video	−0.637	**(−1.287)–(−0.129)**	−0.368	(−0.900)–(0.160)
		Video only	−0.620	**(−1.337)–(−0.062)**	0.005	(−0.663)–(0.745)

Note. 95% CI = 95% confidence interval. Bold = 95% CI did not include 0.

**Table 2:** Results of parameter estimations when the dependent variable was the rating for participants’ perception of the health impact of pit latrines.

Variable	Age	Intervention	After_Environment		Later_Environment	
			Median	95% CI	Median	95% CI
before_environment	Younger	AR + Video	0.337	**(0.013)–(0.725)**	0.292	(−0.090)–(0.832)
		Video only	0.385	**(0.033)–(0.797)**	0.282	(−0.101)–(0.683)
	Older	AR + Video	0.415	**(0.085)–(0.797)**	0.010	(−0.364)–(0.379)
		Video only	0.470	**(0.096)–(0.904)**	0.313	(−0.077)–(0.732)
quiz	Younger	AR + Video	−0.773	**(−1.317)–(−0.219)**	−0.654	**(−1.456)–(−0.063)**
		Video only	−0.742	**(−1.305)–(−0.154)**	−0.202	(−0.897)–(0.459)
	Older	AR + Video	−0.695	**(−1.249)–(−0.160)**	−0.197	(−0.740)–(0.345)
		Video only	−0.727	**(−1.317)–(−0.125)**	0.128	(−0.775)–(0.500)

Note. 95% CI = 95% confidence interval. Bold = 95% CI did not include 0.

**Table 3:** Results of statistical analysis of the participants’ impressions of the information presented.

Perspective	*W*	*p*	*r*
(1) Impressions regarding toilet conditions in developing countries in Asia	404	0.492	0.13
(2) Reality regarding toilet conditions in developing countries in Asia	384	0.330	0.18
(3) Receptiveness to knowledge about toilet conditions in developing countries in Asia	458	0.910	0.02
(4) Willingness to learn more about toilet conditions in developing countries in Asia	441	0.899	0.02
(5) Implications for the pit latrine system	540	0.174	0.25
(6) Concentration when viewing the material	488	0.568	0.11
(7) Enjoyment of the material	658	0.002 ***	0.56

*** *p* < 0.01

## Data Availability

The data are provided as a Supplementary Document.

## Supplementary Materials

The following supporting information can be downloaded at: https://www.mdpi.com/article/10.3390/ijerph21121634/s1, Figure S1: The textual information; Figure S2: Screenshots from the video.
