# Supplementary material for: Raising Awareness for Sustainable Faecal Treatment Using Augmented Reality
Source: Int J Environ Res Public Health. 2024 Dec 8;21(12):1634. doi: 10.3390/ijerph21121634 (PMC11675113; doi:10.3390/ijerph21121634)
Supplement: Supplementary file 1 [file ijerph-21-01634-s001.zip › Figure S1.pdf]

Figure S1 The textual information.

Difference of toilet wastewater treatment

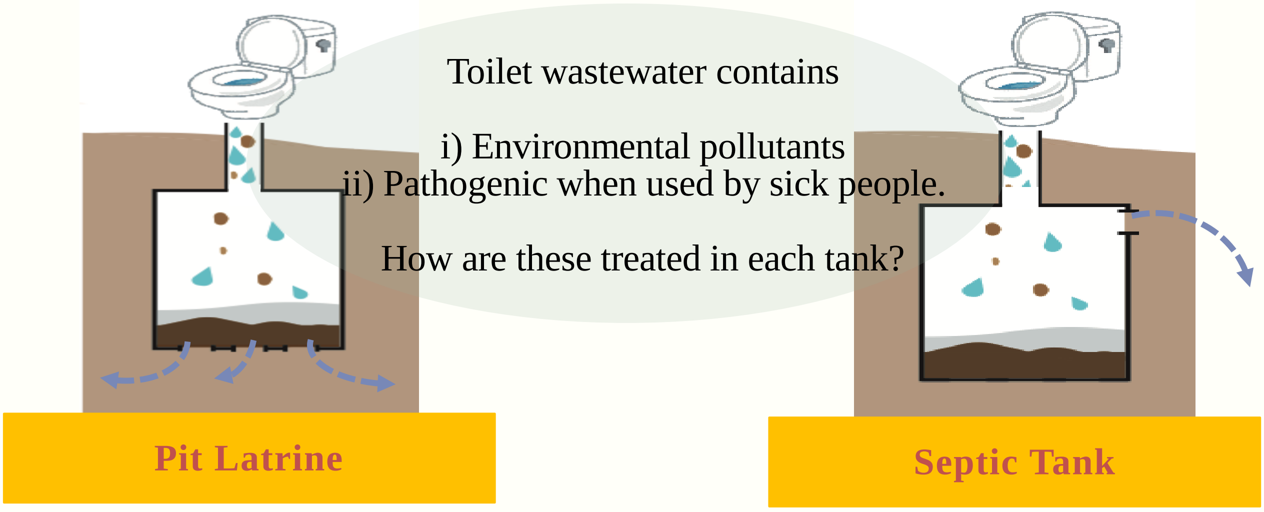

① Stay in the tank for a certain time<br>→Decomposed by microorganisms in the tank・Adsorbed by soil		
**Very short**<br>(A few days)	Staying time	**Long time**<br>Ex)family of 3 with a 3m³ tank: Approx.43 days

Sometimes inadequately treated

runoff from the bottom **runoff from the Top**

②decomposed by microorganisms in the soil・adsorbed in the soil

often do not do	Maintenance	sludge have to be collected every 3~4 years(for a fee)

Dense residential area

⬆ wastewater flowing into the soil
⬆ risk of outbreaks of disease somewhere in the region
→Pathogenic microorganisms enter the toilet hole

⚠ difficult to remove efficiently	processed enough in a long staying time.

→Environmental damage due to groundwater pollution
(bad odor, deterioration of water quality in rivers, etc.)
Health hazard (diarrhea, etc.)
