# Supplementary figures and images for: Raising Awareness for Sustainable Faecal Treatment Using Augmented Reality
Source: Int J Environ Res Public Health. 2024 Dec 8;21(12):1634. doi: 10.3390/ijerph21121634 (PMC11675113; doi:10.3390/ijerph21121634)

Figure S2 Screenshots from the video.

Pit Latrine

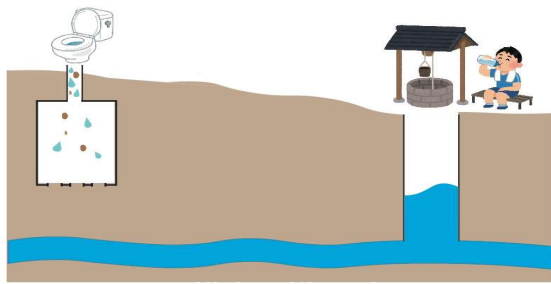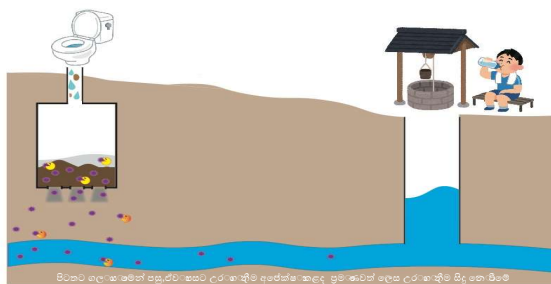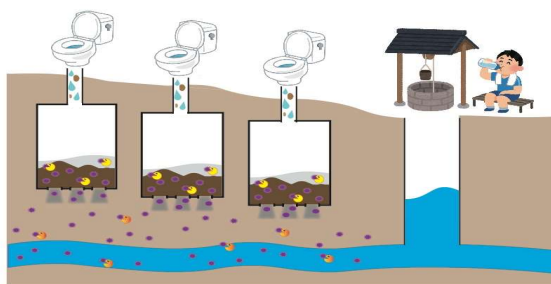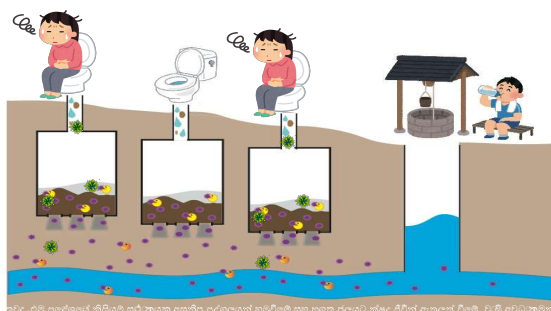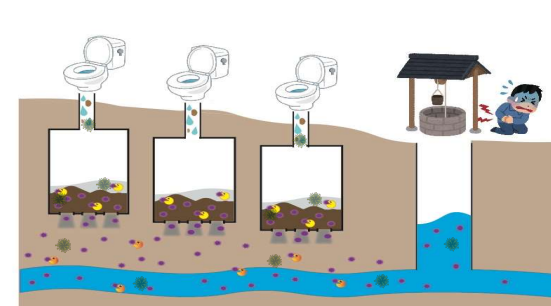

Septic Tank

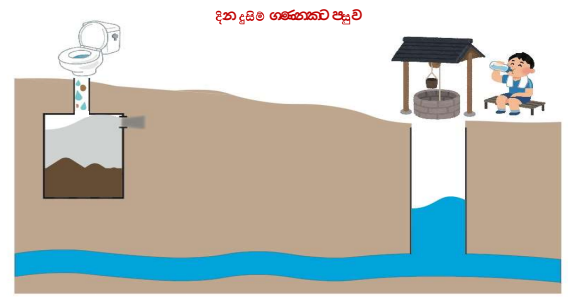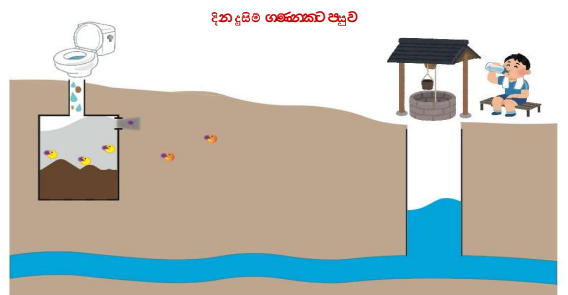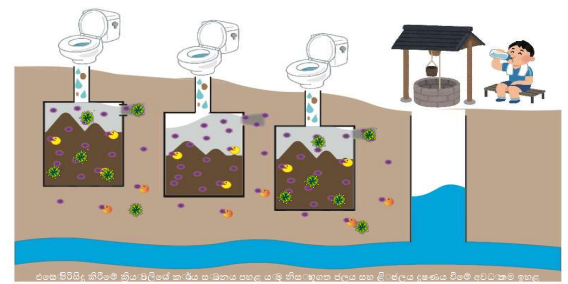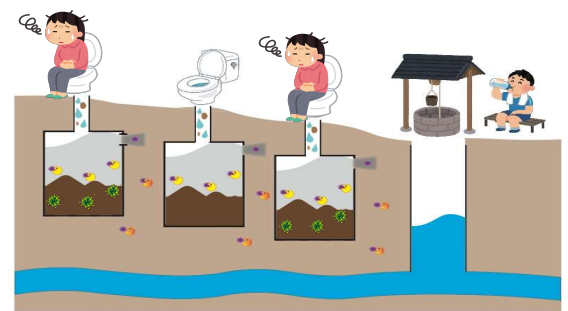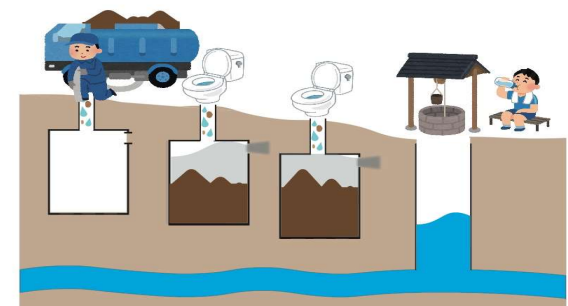

Supplement: Supplementary file 1 [file ijerph-21-01634-s001.zip › Figure S2.pdf]
